# Supplementary material for: Identification of Freezing-Responsive microRNAs and Their Targets in Chinese Jujube by Small RNA and Degradome Sequencing
Source: Int J Mol Sci. 2026 Sep 4;27(17):7912. doi: 10.3390/ijms27177912 (PMC13566890; doi:10.3390/ijms27177912)
Supplement: Supplementary file 1 [file ijms-27-07912-s001.zip › Supplementary Material S2/novel_miR_189.pdf]

Provisional ID	:	novel_miR_189
Score total	:	0.8
Score for star read(s)	:	-1.3
Score for read counts	:	0
Score for mfe	:	0.5
Score for randfold	:	1.6
Score for cons. seed	:	
Total read count	:	87
Mature read count	:	87
Loop read count	:	0
Star read count	:	0

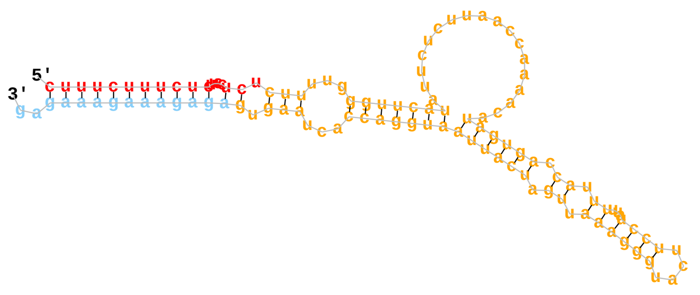

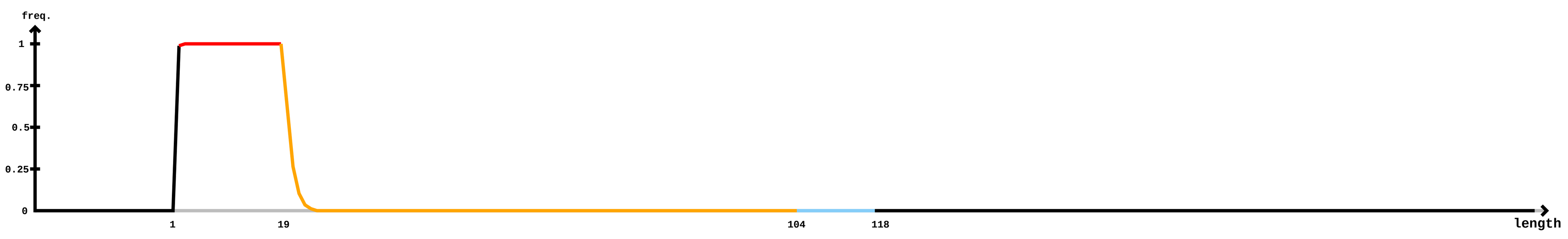


```
                      Mature                                                                                    Star
5'- ucaucaauccuuagccuuuuuucuuucuuucucuuccucucuuuuggguucauauucucuuaaccaaaacauagugaccauuuuuauccuucaugggaaauugaucauuaauggaccacuaagugagagaaagaaagagcucaccaugauauaaccugacuuggguguuuaaaggggguguuuaagggugcaugcuagcgaucucguuggcgucaccuuaacaugguuuuuuguuuguuguagaucagcga   -3'   exp
    ...........((((....((((((((((((....((.(((...(((((((.................((((((.((.((...((((....)))))).)).)))))))))))))...))).)))))))))))))))..((((........((((.....))))........))))....((((((.(((((((((....)))))))))))))))).....))))......((((((.....))))))   reads   mm   sample
    ......................cuuucuuucucuuccucu........................................................................................................   4   0   JCK-2
    ......................cuuucuuucucuuccucu........................................................................................................   8   0   JCK-3
    ......................cuuucuuucucuuccuUu........................................................................................................   2   1   LT-3
    ......................cuuucuuucucuuccucu........................................................................................................   9   0   LT-1
    ......................cuuucuuucucuuccucu........................................................................................................   4   0   JCK-1
    ......................cuuucuuucucuuccucu........................................................................................................   6   0   JT-2
    ......................cuuucuuucucuuccucu........................................................................................................   7   0   LT-3
    ......................cuuucuuucucuuccuUu........................................................................................................   2   1   JCK-1
    ......................cuuucuuucucuuccuUu........................................................................................................   1   1   JCK-2
    ......................cuuucuuucucuuccuUu........................................................................................................   2   1   JCK-3
    ......................cuuucuuucucuuccucu........................................................................................................   4   0   LCK-3
    ......................cuuucuuucucuuccucu........................................................................................................   9   0   JT-3
    ......................cuuucuuucucuuccuAuc.......................................................................................................   6   1   LCK-3
    ......................cuuucuuucucuuccucuUu......................................................................................................   1   1   LT-2
    ......................cuuucuuucucuuccucuUu......................................................................................................   2   1   LT-3
    ......................cuuucuuucucuuccucuUu......................................................................................................   7   1   JCK-2
    ......................cuuucuuucucuuccucucu......................................................................................................   1   0   JCK-2
    ......................cuuucuuucucuuccucuUu......................................................................................................   2   1   JCK-3
    ......................cuuucuuucucuuccucuAu......................................................................................................   1   1   LT-3
    ......................cuuucuuucucuuccucuUuu.....................................................................................................   1   1   LT-3
    ......................cuuucuuucucuuccucuAuu.....................................................................................................   1   1   LT-3
    ......................cuuucuuucucuuccucuAuu.....................................................................................................   4   1   JCK-2
    ......................cuuucuuucucuuccucuUuuu....................................................................................................   2   1   LT-2
    .......................uuucuuucucuuccucuUuuuu...................................................................................................   1   1   LT-1
```
